# Supplementary material for: Remodeling tumor microenvironment by liposomal codelivery of DMXAA and simvastatin inhibits malignant melanoma progression
Source: Sci Rep. 2021 Nov 11;11:22102. doi: 10.1038/s41598-021-01284-5 (PMC8585864; doi:10.1038/s41598-021-01284-5)
Supplement: Supplementary file 1 — Supplementary Figures. [file 41598_2021_1284_MOESM1_ESM.pdf]

Supplementary information

Remodeling tumor microenvironment by liposomal codelivery of DMXAA and simvastatin inhibits malignant melanoma progression

Valentin-Florian Rauca[1,2], Laura Patras[1], Lavinia Luput[1], Emilia Licarete[1,3], Vlad-Alexandru Toma[1,4,5] Alina Porfire[6], Augustin Catalin Mot[7], Elena Rakosy-Tican[1], Alina Sesarman[1*], Manuela Banciu[1]

Author/co-authors contact details

Valentin-Florian Rauca: valentin.rauca@tum.de
Laura Patras: patras.laura88@yahoo.com
Lavinia Luput: lavinia_luk@yahoo.com
Emilia Licarete: emilia_licarete@yahoo.com
Vlad-Alexandru Toma: tomavlad91@yahoo.com
Alina Porfire: aporfire@umfcluj.ro
Augustin Catalin Mot: augustin.mot@ubbcluj.ro
Elena-Rakosy-Tican: elena.rakosy@ubbcluj.ro
Alina Sesarman: sesarman@gmail.com* - corresponding author
Manuela Banciu: manuela.banciu@ubbcluj.ro

Affiliations

[1]Department of Molecular Biology and Biotechnology, and Center of Systems Biology, Biodiversity and Bioresources, Faculty of Biology and Geology, Babes-Bolyai University, 5-7 Clinicilor Street, 400006 Cluj-Napoca, Romania.
[2]Department of Dermatology and Allergy, School of Medicine, Technical University of Munich, 29 Biedersteiner Street, 80802 Munich, Germany.
[3]Molecular Biology Centre, Institute for Interdisciplinary Research in Bio-Nano-Sciences of Babes-Bolyai University, 42 Treboniu Laurian Street, 400271 Cluj-Napoca, Romania.
[4]Department of Experimental Biology and Biochemistry, Institute of Biological Research, Branch of NIRDBS Bucharest, 48 Republicii Street, 400015 Cluj-Napoca, Romania.
[5]Department of Molecular and Biomolecular Physics, National Institute of Research and Development for Isotopic and Molecular Technologies, 67-103 Donath Street, 400293 Cluj-Napoca, Romania.
[6]Department of Pharmaceutical Technology and Biopharmaceutics, Faculty of Pharmacy, University of Medicine and Pharmacy "Iuliu Hatieganu", 8 Babes Street, 400012 Cluj-Napoca, Romania.
[7]Research Center for Advanced Chemical Analysis, Instrumentation and Chemometrics, Faculty of Chemistry and Chemical Engineering, Babes-Bolyai University, 11 Arany Janos Street, 400028 Cluj-Napoca, Romania.

Contact information for corresponding author: Alina Sesarman, PhD
Department of Molecular Biology and Biotechnology,
Faculty of Biology and Geology, Babes-Bolyai University,
5-7, Clinicilor Street, 400006, Cluj-Napoca, Romania
Tel: +40264431691; Mobile:+40752075505; Fax: +40264431858.
Email: sesarman@gmail.com;

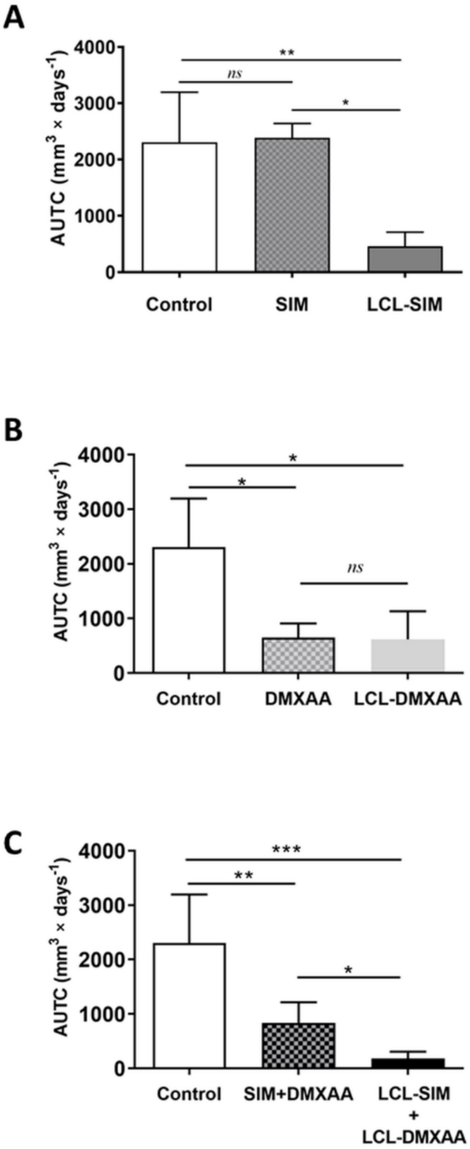

Supplementary Figure 1: AUTC in C57BL/6 mice treated with liposomal SIM and DMXAA. Mice received two *i.v.* injections of therapeutic agents at day 11 and day 14, after cancer cell inoculation. AUTC was measured until day 15 in melanoma bearing mice after various treatments with free or liposomal SIM **(A),** free or liposomal DMXAA **(B),** and after free or liposomal SIM+DMXAA **(C).** Control – LCL-treated group; SIM – experimental group treated with 5 mg/kg free SIM; LCL-SIM – experimental group treated with 5 mg/kg SIM as liposome-encapsulated form; DMXAA – experimental group treated with 14 mg/kg free DMXAA; LCL-DMXAA – experimental group treated with 14 mg/kg DMXAA as liposome-encapsulated form; SIM+DMXAA – experimental group treated with 5 mg/kg free SIM and 14 mg/kg free DMXAA; LCL-SIM + LCL-DMXAA – experimental group treated with 5 mg/kg SIM and 14 mg/kg DMXAA as liposome-encapsulated forms. Results were expressed as mean ± SD of tumor volumes and each experimental group consisted of 5 mice. (*ns*, $P>0.05$; *, $P<0.05$;

**, $P<0.01$; ***, $P<0.001$). The graphs were generated using GraphPad Prism 9.2.0.332 (Serial number: GPS-2216002-E###-#####, MachineID: 3383874FBDD)(https://www.graphpad.com/)

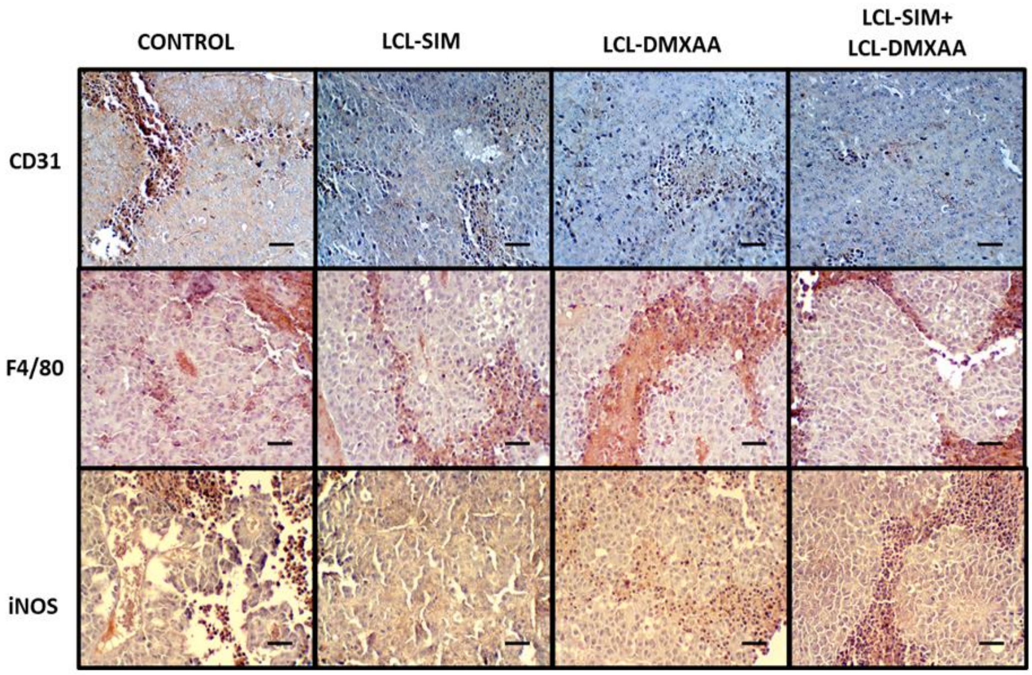

Supplementary Figure 2. Immunohistochemical analysis of tumor tissues from B16.F10 melanoma bearing mice treated with liposomal SIM or/and DMXAA. Positively stained cells for CD31, a marker for proliferating endothelial cells, F4/80 an antigen expressed on murine macrophages and iNOS, an M1 macrophages marker, appear in brown; size bars = 50 μm. Images were processed using open source IrfanView graphic viewer 64-bit Version 4.58 (https://www.irfanview.com/) and multi-panel figure assembly was generated using Microsoft® Office Home and Student 2019 MSO 64-bit (product ID: 00405-57743-39916-AA493) (https://www.microsoft.com/)

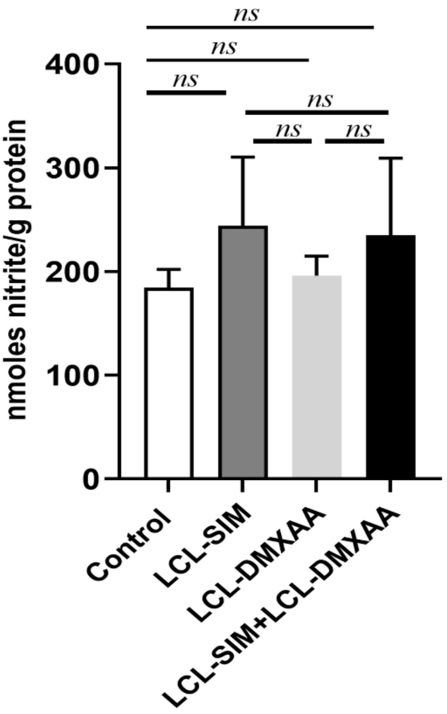

Supplementary Figure 3. Effects of liposome-encapsulated SIM or/and DMXAA on intratumor levels of nitrite. The results are expressed as mean ± SD of two independent measurements. One way ANOVA test with Bonferroni correction for multiple comparisons was performed to analyze the differences between experimental conditions (*ns*, $P>0.05$). Control – LCL-treated group; LCL-SIM – experimental group treated with 5 mg/kg SIM as liposome-encapsulated form; LCL-DMXAA – experimental group treated with 14 mg/kg DMXAA as liposome-encapsulated form; LCL-SIM + LCL-DMXAA – experimental group treated with 5 mg/kg SIM and 14 mg/kg DMXAA as liposome-encapsulated forms. The graph was generated using GraphPad Prism 9.2.0.332 (Serial number: GPS-2216002-E###-#####, MachineID: 3383874FBDD)(https://www.graphpad.com/)

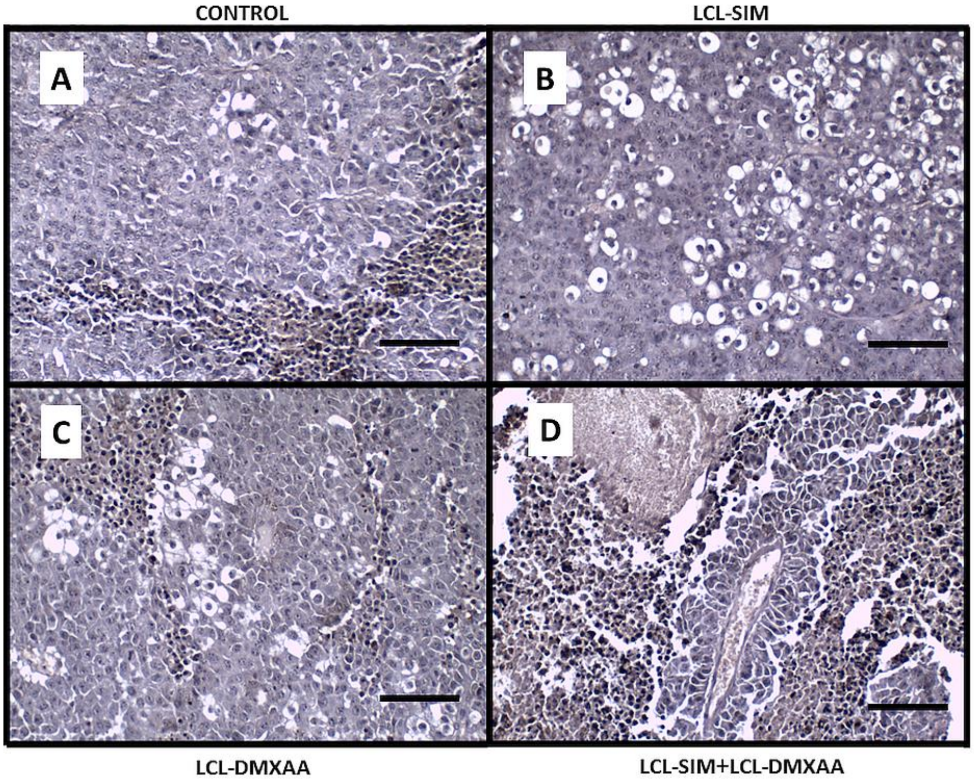

Supplementary Figure 4. Histopathological evaluation of the effects of different treatments on B16.F10 murine melanoma microenvironment in vivo. Tissue sections were stained by hematoxylin- eosin method for histological examination; Size bars = 10 μm. **(A)** uncompact histological feature characterized by mitotic nuclei and negligible cytoplasmic vacuolation in tissue sections from control group; **(B)** intense karyorrhexis and abundant cytoplasmic vacuolation in LCL-SIM-treated tumors; **(C)** moderate inflammatory infiltrate and cytoplasmic vacuolation in LCL-DMXAA treated group**; (D)** intense karyolysis, hyperchorome nuclei and pericapillary infiltration in LCL-SIM+LCL-DMXAA group. Images were processed using open source IrfanView graphic viewer 64-bit Version 4.58 (https://www.irfanview.com/) and multi-panel figure assembly was generated using Microsoft® Office Home and Student 2019 MSO 64-bit (product ID: 00405-57743-39916-AA493) (https://www.microsoft.com/).

Full length blots for representative cropped western blot images, presented in the manuscript

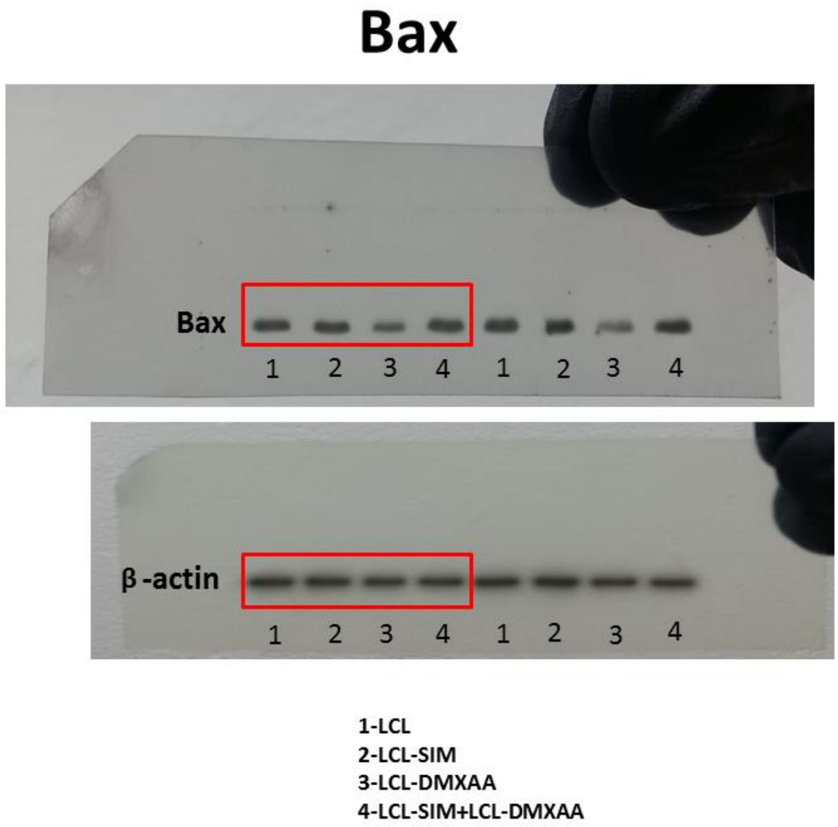

Figure 4. Original images used in panel A, showing Bax and β-actin expression

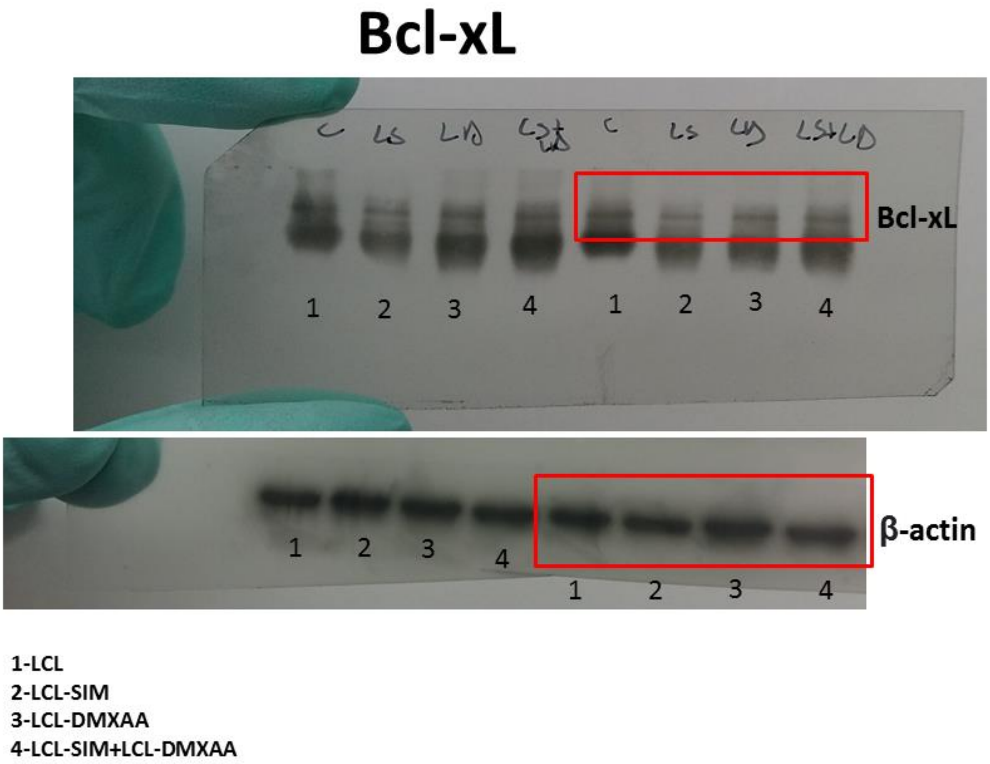

Figure 4. Original images used in panel B, showing Bcl-xL and β-actin expression

HIF-1α

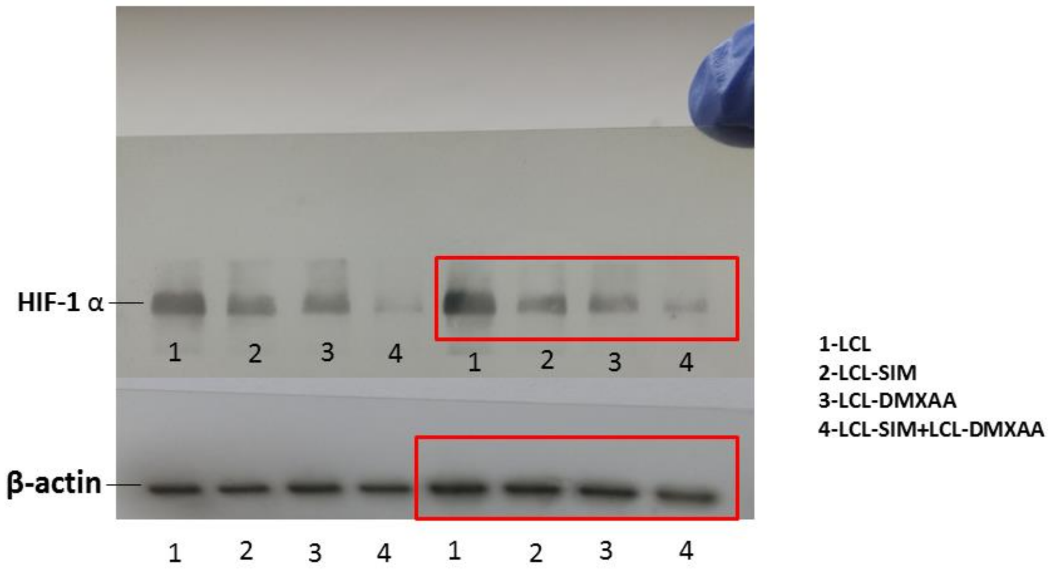

Figure 6. Original images used in panel A, showing HIF-1α and β-actin expression

pAP1-c-Jun

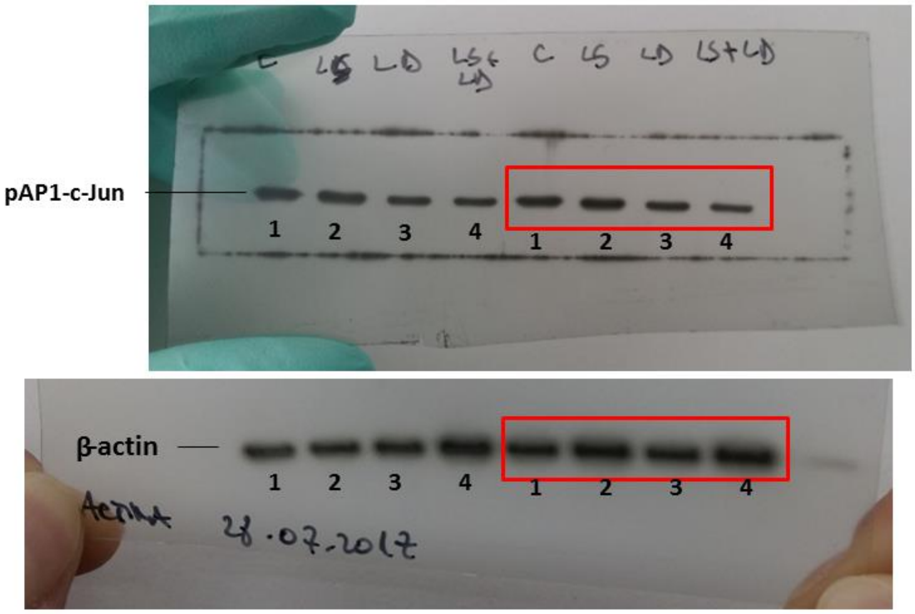

1-LCL
2-LCL-SIM
3-LCL-DMXAA
4-LCL-SIM+LCL-DMXAA

Figure 6. Original images used in panel C, showing pAP-1 c-Jun and β-actin expression

MMP-2, MMP-9

1-FREE
2-SIM
3-DMXAA
4-SIM+DMXAA
5- LCL
6- LCL-SIM
7-LCL-DMXAA
8-LCL-SIM+LCL-DMXAA
M- marker

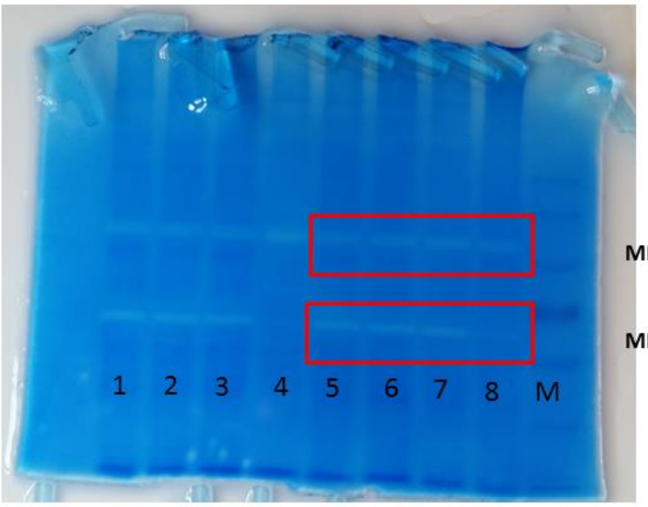

MMP-9

MMP-2

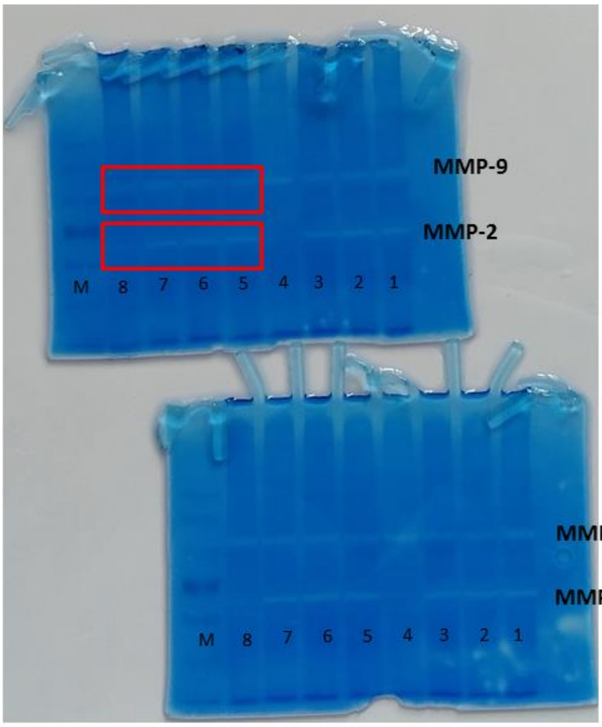

1-FREE
2-SIM
3-DMXAA
4-SIM+DMXAA
5- LCL
6- LCL-SIM
7-LCL-DMXAA
8-LCL-SIM+LCL-DMXAA
M- marker

Figure 6. Original images used in panel E (2 replicates of the zymographic gels), showing MMPs activity
